# Cutaneous Mucormycosis Complicating a Polymicrobial Wound Infection Following a Dog Bite

**DOI:** 10.1155/2011/348046

**Published:** 2011-07-03

**Authors:** Dalila Zachary, Kimberly Chapin, Linda Binns, Karen Tashima

**Affiliations:** ^1^Warren Alpert Brown University School of Medicine, 1125 North Main Street, Providence, RI 02906, USA; ^2^Warren Alpert Brown University School of Medicine, 593 Eddy Street APC 12, Providence, RI 02903, USA; ^3^Rhode Island Hospital, 593 Eddy Street APC 12, Providence, RI 02903, USA

## Abstract

We report a case of cutaneous mucormycosis and *Enterobacter* infection developing in a 50-year-old diabetic woman following a dog bite that showed delayed development and diagnosis in comparison with typical zygomycotic cutaneous lesions.

## 1. Introduction 

The zygomycetes are a class of fungi that are ubiquitous in soil and decaying vegetable matter but can cause a variety of infections in humans. The genera most commonly found in human infections are *Rhizopus *and *Mucor *[1]. Infection typically occurs in immunosuppressed patients such as those with transplants, malignancies, and diabetes mellitus and is usually associated with rhino-orbital-cerebral and pulmonary disease [2]. Infection of the skin and soft tissues with zygomycetes is the third most common diagnosis after sinus and pulmonary forms [1]. Cutaneous zygomycosis results from inoculation of the spores into disrupted dermis and can occur in patients with little or no underlying immunodeficiency [3–5].

## 2. Case Report 

**Figure 1:**
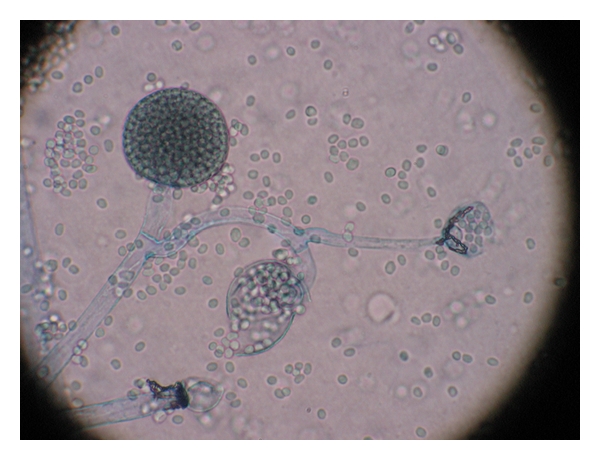
Microscopic sample obtained from patient's wounds. It shows presence of *mucor sp. *

A 50-year-old woman was referred to the Infectious Disease Clinic for nonhealing painful hand wounds, twelve days after sustaining dog bites on the dorsum of both hands. She had a history of insulin-dependent diabetes mellitus for two years and hypertension. She was initially evaluated at an urgent care center in Rhode Island on the day of the dog bite. At that time, she was noted to have open wounds on the dorsum of both hands which were subsequently cleaned and stitched. She was given a fourteen-day course of amoxicillin. Six days after the initial dog bite, she returned to the urgent care center to have the stitches removed. At that time, she was noted to have an enlarged, draining nodule on the left hand. As a result, the stitches were only removed from the right hand. The bloody drainage from the left hand wound was sent for culture. Three days later, nine days after initial dog bite, she was called back to the urgent care center and was told mold and bacteria grew in the culture and that the mold was likely a contaminant. At this visit, a small eschar replaced the draining nodule on the dorsum of her left hand. The stitches were removed from the left hand and a black, stringy material was noted in the wound. This material was sent to the microbiology laboratory for culture, and the wound was packed. The patient continued her amoxicillin and eleven days after the dog bite, she returned to the urgent care center for the fourth time and once again was told that the culture grew the same bacteria and mold, which was later identified as *Enterobacter cloacae* and *Mucor* species (Figure 1). The patient was referred to the Infectious Disease Clinic. 

**Figure 2:**
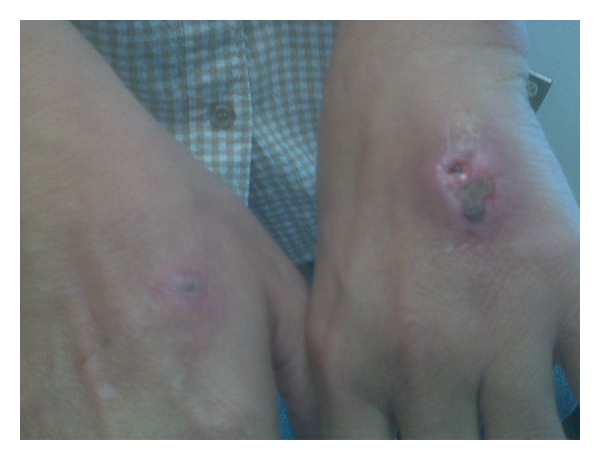
Ulcerative, nodular wounds on the dorsum of bilateral hands where patient sustained dog bites. Left hand is worse than right.

The patient denied any other unusual exposures following the dog bites. She denied fever or chills. Her current medications were insulin, metformin, aspirin, and lisinopril. She had no known drug allergies. At the Infectious Disease Clinic, her exam was remarkable for normal vital signs and bilateral hand ulcers, left greater than right (Figure 2). The patient was sent from the Infectious Disease Clinic to the Emergency Department (ED) in order to be evaluated for debridement. We recommended that the patient receive meropenem 1 mg/kg and 5 mg/kg of amphotericin B (lipid complex; Abelcet; Enzon Pharmaceuticals, Bridgewater, NJ, USA). 

**Figure 3:**
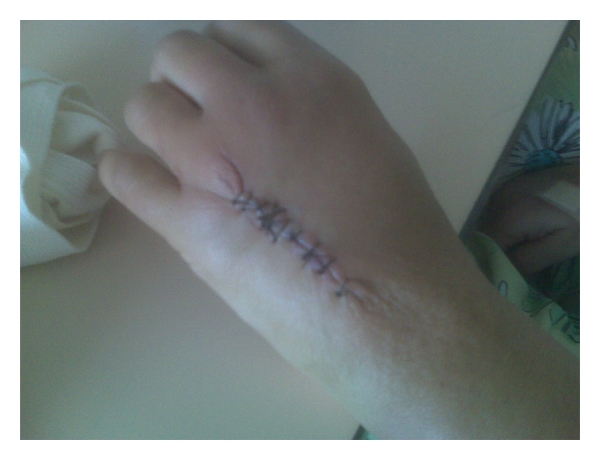
Patient's left hand following surgical debridement.

In the ED, the patient's white blood cell (WBC) was 7900/uL, with a normal differential and normal chemistry panel. Her hemoglobin A1c was 7.8%. Blood cultures were drawn, which were negative for growth. Within three hours of arrival to the ED, the patient was taken to the Operating Room (OR) for debridement of left dorsal hand wound. The surgeons debrided down through the subcutaneous tissue to the level of the peritenon, which are the connective tissue structures attached to and surround the tendon (Figure 3). The specimen was sent to pathology and microbiology. This time, the cultures did not grow. The pathologist found chronic active ulcer and organizing abscess in the surgical specimen. 

Three days after surgical debridement, the patient's wound was healing well, and her intravenous antibiotics were switched to ciprofloxacin 500 mg orally every twelve hours for two weeks and posaconazole 100 mg orally every twelve hours for two weeks.

## 3. Discussion 

The presence of more than one organism isolated from a wound following a dog bite is not unusual, but the presence of *Mucor *sp is atypical. The most common bacteria found in a dog's mouth include: *Staphylococcus* species, *Streptococcus* species, *Eikenella* species, *Pasteurella* species, *Proteus* species, *Klebsiella* species, *Enterobacter* species, and *Capnocytophaga canimorsus* [6]. Thus, the *Enterobacter* infection likely came from bacteria already in the dog's mouth. We postulate that this patient's fungal infection may have resulted from the dog's mouth being contaminated with soil that contained *Mucor *sp. Alternatively, the patient could have contaminated her wound with soil from her yard. However, the latter seems less likely, as the patient did not recall putting her hands in soil after the dog bite. Infection with zygomycetes has also been associated with contaminated traumatic wounds, dressings and splints, burns, and surgical sites [3–5]. This case is significant, because it is difficult to find case reports in the literature of polymicrobial infection involving *Mucor* sp. following a dog bite. Additionally, previous case reports of cutaneous zygomycosis involve patients with greater immunosuppression [7, 8] although cutaneous mucormycosis can occur in patients with little or no immunodeficiency [9]. Our patient had diabetes, but her glucose level was moderately controlled with a hemoglobin A1c less than 8%. 

The development and diagnosis of cutaneous zygomycosis was also delayed in this case compared to its more rapid development in immunosuppressed patients [7, 8]. The diagnosis is often made on clinical grounds and can be very challenging; tissue biopsy for histology and culture is usually needed [3]. The gross appearance of skin lesions infected with *Mucor* sp. varies but most often causes an ulcerative lesion with eschar formation [10], as it did in our patient. Lesions consistent with cutaneous zygomycosis were first noted 9 days after the patient's dog bite. The first time *Mucor* sp was isolated from our patient, it was thought to be a contaminant and thus was not treated immediately, causing a delay in diagnosis and appropriate treatment. Finding the same organism twice did prompt medical personnel to reconsider the diagnosis of fungal infection in this patient who was not responding to appropriate medical therapy. 

The best outcomes from cutaneous zygomycosis have been associated with early detection, aggressive surgical debridement, early use of effective antifungal therapy, and correction of predisposing factors [11]. Although intravenous amphotericin B (lipid formulation) is the drug of choice initially, oral posaconazole is used as step-down therapy for patients who have responded to amphotericin B [12]. Despite early diagnosis and aggressive combined surgical and medical therapy, the prognosis for recovery from zygomycosis is poor with the exception of cutaneous involvement, which rarely disseminates [11]. First-line oral antibiotic therapy following a dog bite is amoxicillin-clavulanic acid for 10 days, which was appropriately given to the patient. Unfortunately, amoxicillin-clavulanic acid is not effective against mold, and thus did not treat our patient's fungal infection. Diagnosis of *Mucor* sp as a pathogen in the wound infection would have resulted in a more appropriate antibiotic earlier in her treatment course.

## 4. Conclusion

In conclusion, this case highlights the importance of diligence in collecting microbiologic data when there is minimal clinical response to empiric antibiotics. In any patient, where there is a possibility of soil contamination and the presence of an advancing necrotic indurated lesion, consideration of zygomycosis as the diagnosis should be made. It is important to consider the microbiologic results before dismissing them as contamination and recognize mold as an important and potential pathogen. The patient was taking amoxicillin for several days without clinical improvement, and it became critical that cultures were taken in order to establish the diagnosis. Lastly, this patient received a referral from the urgent care center to an infectious disease specialist and had a surgical evaluation and debridement. Because all of these critical steps (obtaining cultures, infectious disease, and surgical consultations) were taken during the care of this patient, she had a good outcome.
